# Development and validation of a nomogram for predicting postoperative bone nonunion in spinal tuberculosis patients

**DOI:** 10.3389/fsurg.2026.1753872

**Published:** 2026-04-13

**Authors:** Wentao Zhao, Yongrui Yang, Wenkai Run, Jianlong Li, Rongpan Dang, Huigang An, Liang Xu, Yingxin Zhao, Hongdong Tan

**Affiliations:** Department of Orthopedics, Public Health Clinical Center Affiliated to Shandong University, Jinan, China

**Keywords:** bone nonunion, nomogram, radiology, spinal tuberculosis, spondylitis

## Abstract

**Background:**

Postoperative bone nonunion is a critical complication following instrumented fusion for spinal tuberculosis. Preoperative prediction is essential for prevention. While clinical risk factors exist, current predictive tools lack validation in infected cohorts.

**Purpose:**

This study developed and validated a multivariate nomogram, provided an individualized preoperative estimate of nonunion risk in spinal tuberculosis patients, incorporating key clinical and radiological predictors to guide preventative strategies.

**Method:**

A retrospective cohort of 178 patients undergoing debridement and instrumented fusion for spinal tuberculosis (Shandong Public Health Clinical Center, January 2021-January 2024) was stratified by Bridwell classification into union (*n* = 120) and nonunion (*n* = 58) groups. Perioperative variables were compared between groups. Predictive features were selected via least absolute shrinkage and operator selection (LASSO) regression and incorporated into a multivariate logistic regression model. A nomogram was constructed based on the model. Calibration was assessed using the Hosmer-Lemeshow test with calibration curves, and discriminative ability was evaluated by the area under the ROC curve (AUC). Decision curve analysis (DCA)was performed to estimate the clinical usefulness of the prediction model by quantifying the net benefits at different threshold probabilities.

**Results:**

The training cohort of this study comprised 178 patients, of which 120 presented with union and 58 with nonunion. Five predictor variables were screened by LASSO regression and plotted as a nomogram, including ALB, CRP normalization days, Bone graft materials, Psoas abscess, Jumping lesions. The nomogram showed strong discrimination and solid calibration, AUC = 0.947 (95% confidence 0.915–0.978). The calibration curves of the diagnostic models showed the optimal concordance between the predicted results and the actual observations. The DCA indicated that the substantial clinical net benefit across threshold probabilities.

**Conclusion:**

The study successfully developed a precise and effective nomogram for identifying postoperative bone nonunion in spinal tuberculosis patients. This nomogram aids early detection and prevention in postoperative bone nonunion, improving clinical decisions and treatment optimization.

## Introduction

Spinal tuberculosis (STB) constitutes a severe and challenging form of extrapulmonary tuberculosis (1, 2). Its management is particularly difficult due to the destructive nature of the infection, often necessitating surgical intervention when conservative treatments fail or neurological deficits emerge. Surgical strategies, including extensive debridement and instrumented spinal fusion, are critical for eradicating the infection, reconstructing spinal stability, and decompressing neural structures (2–5). Despite improvements in surgical techniques, anti-tuberculosis chemotherapy, and perioperative care, the treatment course remains protracted and fraught with challenges. The surgery itself is technically demanding due to pre-existing vertebral destruction, complex deformities, and the avascular nature of infected tissues. Moreover, postoperative bone nonunion remains a common and serious complication, often leading to implant failure, deformity recurrence, and poor functional outcomes. Although several tools have been proposed to predict nonunion in general surgeries, these models are limited by small sample sizes and lack validation in STB-specific populations (6, 7). Therefore, a reliable predictive tool tailored to nonunion risk after STB surgery remains an unmet clinical need.

Nomograms have emerged as valuable clinical tools for individualizing risk prediction (8–12). These user-friendly graphical calculators integrate multiple independent predictive variables, each assigned a weighted score, to estimate the probability of a specific clinical outcome for an individual patient. While nomograms have been developed for various outcomes in spinal surgery, a validated nomogram specifically designed to predict postoperative bone nonunion risk in patients undergoing surgery for active spinal tuberculosis, incorporating both comprehensive clinical variables and pertinent radiological biomarkers, is currently lacking.

Therefore, this study aimed to address this significant clinical gap. We sought to develop and rigorously validate a novel, multivariate predictive nomogram for postoperative bone nonunion in patients undergoing debridement and instrumented fusion for spinal tuberculosis. The resulting nomogram was designed to provide surgeons with a practical, evidence-based tool for precise preoperative risk stratification.

## Materials and methods

Consecutive patients with clinically and/or pathologically confirmed STB treated between Jan 2021 and Jan 2024 at the Shandong Public Health Clinical Center, were included as the training cohort and the internal validation cohort. This retrospective study was approved by the Institutional Review Board of Shandong Public Health Clinical Center. The requirement for informed consent was waived due to the retrospective nature of the analysis. The inclusion criteria for this study were as follows: (1) Definitive diagnosis of spinal tuberculosis based on clinical presentation, laboratory markers, microbiological culture (blood, CT-guided biopsy, or intraoperative tissue), and characteristic imaging findings (MRI/CT). (2) Posterior surgical approach involving radical debridement and instrumented fusion with pedicle screw fixation. (3) Availability of complete preoperative clinical, laboratory, and radiological data. (4) Minimum follow-up of 12 months with CT scans to assess fusion status. Exclusion criteria were (1) Patients with infectious elsewhere in the body (2) Active malignancy or metastatic spinal disease (3) Pathological fractures unrelated to infection. (4) Previous fusion surgery at the same spinal level. (5) Incomplete follow-up data or imaging. A total of 118 patients were excluded because of other infections and non-infectious lesions. This study finally included 178 patients, with union group (*n* = 120) and nonunion group (*n* = 58) according to Bridwell grade.

Bone fusion status was evaluated at the final follow-up using computed tomography (CT) scans and classified according to the Bridwell fusion grading system. Grades I and II were defined as successful fusion, whereas Grades III and IV were considered bone nonunion (13). Fusion status was independently assessed by two investigators blinded to clinical outcomes, and discrepancies were resolved by consensus with a senior spine surgeon.

Preoperative, intraoperative, and postoperative data were systematically extracted from electronic medical records (EMR) and Picture Archiving and Communication Systems (PACS) by two independent researchers blinded to the final fusion outcome.

Measures that conformed to a normal distribution were described using the mean ± standard deviation and t-tests for two independent samples were used for comparisons between groups. Measures that did not conform to a normal distribution were described using the median and percentile, and comparisons between groups were made using a non-parametric test. The chi- square test was used for inter-group comparisons of the count data. Predictors were screened using the least absolute shrinkage and operator selection (LASSO) regression technique, and postoperative bone nonunion prediction models and column line plots (nomograms) were developed using multifactorial logistic regression. The calibration curve of the model was plotted using the Hosmer- Lemeshow goodness-of-fit test. The predictive effect of the model was evaluated by calculating the area under the ROC curve (AUC). Decision curve analysis (DCA) was performed to estimate the clinical usefulness of the prediction model by quantifying the net benefits at different threshold probabilities.

To reduce the risk of model overfitting, predictor selection followed a structured process. Variables deemed clinically relevant or significant in univariate analysis were entered into a LASSO regression model. The one-standard-error (1-SE) criterion was applied to identify the most parsimonious set of predictors.

A total of 58 nonunion events were observed, and five variables were retained in the final multivariable model, yielding an events-per-variable (EPV) ratio of approximately 11.6, which meets conventional recommendations for logistic regression modeling (14).

Internal validation was performed using 500 bootstrap resamples to evaluate model stability and optimism-corrected performance. Only patients with complete clinical, laboratory, and radiological data were included; therefore, no imputation for missing data was performed.

Statistical analyses and image plotting were performed using R software (version 4.2.2; R Foundation for Statistical Computing, Vienna, Austria) and differences were considered statistically significant when *p* < 0.05.

## Results

### Baseline characteristics

Between Jan 2021 and Jan 2024, 296 patients were retrospectively screened from Shandong Public Health Clinical Center, 178 of whom met the eligibility criteria and were divided into union group (*n* = 120) and nonunion group (*n* = 58).

The demographic information and clinical characteristics of the participants are shown in Table 1. Among 120 spinal tuberculosis patients in the union group, 60 (50%) were female and 60 (50%) were male. The nonunion group consisted of 58 patients, with 34 (58.6%) females and 24 (41.3%) males. The mean age of the entire study population was 54 (43, 64) years, while union group and nonunion group had mean age of 51 (42, 62) years and 57.5 (45.5, 66.75) years.

**Table 1:** Demographic information and clinical characteristics of the participants for STB cases. DM, diabetes mellitus; AID, autoimmune disease; OP, operation.

Variables	Union group (*n* = 120)	Nonunion group (*n* = 58)	*P*
Gender, *n* (%)			0.358
Female	60 (50)	24 (41)	
Male	60 (50)	34 (59)	
Age, Median (Q1, Q3)	51 (42, 62)	57.5 (45.5, 66.75)	0.016
DM, *n* (%)			0.312
No	108 (84.4)	49 (84.5)	
Yes	20 (15.6)	9 (15.5)	
AID, *n* (%)			0.006
No	117 (98)	50 (86)	
Yes	3 (2)	8 (14)	
Radiating pain, *n* (%)			0.941
No	87 (72)	41 (71)	
Yes	33 (28)	17 (29)	
Abnormal Liver/Renal Function, *n* (%)			1
No	110 (98)	53 (98)	
Yes	10 (8.3)	5 (8.6)	
Site of lesion, *n* (%)			0.134
Cervical vertebrae	4 (3)	1 (2)	
Thoracic vertebrae	80 (67)	31 (53)	
Lumbar vertebrae	36 (30)	26 (45)	
Jumping lesions, *n* (%)			0.009
No	104 (87)	40 (69)	
Yes	16 (13)	18 (31)	
Psoas abscess, *n* (%)			0.001
No	102 (85)	36 (62)	
Yes	18 (15)	22 (38)	
Number of affected vertebral bodies, *n* (%)			0.915
1	7 (6)	2 (3)	
2	97 (81)	47 (81)	
3	12 (10)	7 (12)	
4	4 (3)	2 (3)	
Pedicle destruction, *n* (%)			0.861
No	96 (80)	45 (78)	
Yes	24 (20)	13 (22)	
OP > 3 h, *n* (%)			0.002
No	60 (50)	14 (24)	
Yes	60 (50)	44 (76)	
Bone Grafting Techniques, *n* (%)			0.035
Posterior	82 (68)	49 (84)	
Anterior	38 (32)	9 (16)	
Bone Graft Materials, *n* (%)			<0.001
Autografts	92 (77)	22 (38)	
Synthetic materials	28 (23)	36 (62)	
Post op Drainage, Mean ± SD	485.49 ± 222.99	585.09 ± 300.01	0.027
CRP normalization days, Median (Q1, Q3)	7 (6, 10)	11 (8, 14)	<0.001
HB, Mean ± SD	118.2 ± 17.36	103.91 ± 15.57	<0.001
ALB, Median (Q1, Q3)	36.65 (34.48, 40.42)	29.7 (28.63, 32.38)	<0.001
Pre op CRP, Median (Q1, Q3)	10.25 (4.95, 22.48)	21.6 (13.45, 37.28)	<0.001
Pre op ESR, Median (Q1, Q3)	57 (38, 68)	67 (55, 72)	0.017
Pre op WBC, Median (Q1, Q3)	6.26 (4.96, 7.52)	5.86 (4.89, 6.92)	0.226
HGB, Mean ± SD	118.2 ± 17.36	121.91 ± 15.57	0.154
PLT, Median (Q1, Q3)	257.5 (224.5, 315.25)	262.5 (220, 307.75)	0.635
PCT, Median (Q1, Q3)	0.11 (0.1, 4.17)	0.56 (0.1, 6.88)	0.443
ALT, Median (Q1, Q3)	15 (10.75, 27)	17 (11, 28.75)	0.989
AST, Median (Q1, Q3)	18 (13, 23.25)	16.5 (13.25, 21)	0.609
Prealbumin, Median (Q1, Q3)	168 (120.25, 216.5)	185 (112.75, 221.5)	0.796
BUN, Median (Q1, Q3)	4.66 (3.42, 5.92)	5.01 (4.1, 6.65)	0.128
Cr, Median (Q1, Q3)	54.9 (48.53, 67)	55.3 (48.05, 69.05)	0.805
Blood Glucose, Median (Q1, Q3)	5.24 (4.75, 5.9)	5.15 (4.68, 5.98)	0.425

### Clinical predictor selection

**Figure 1:**
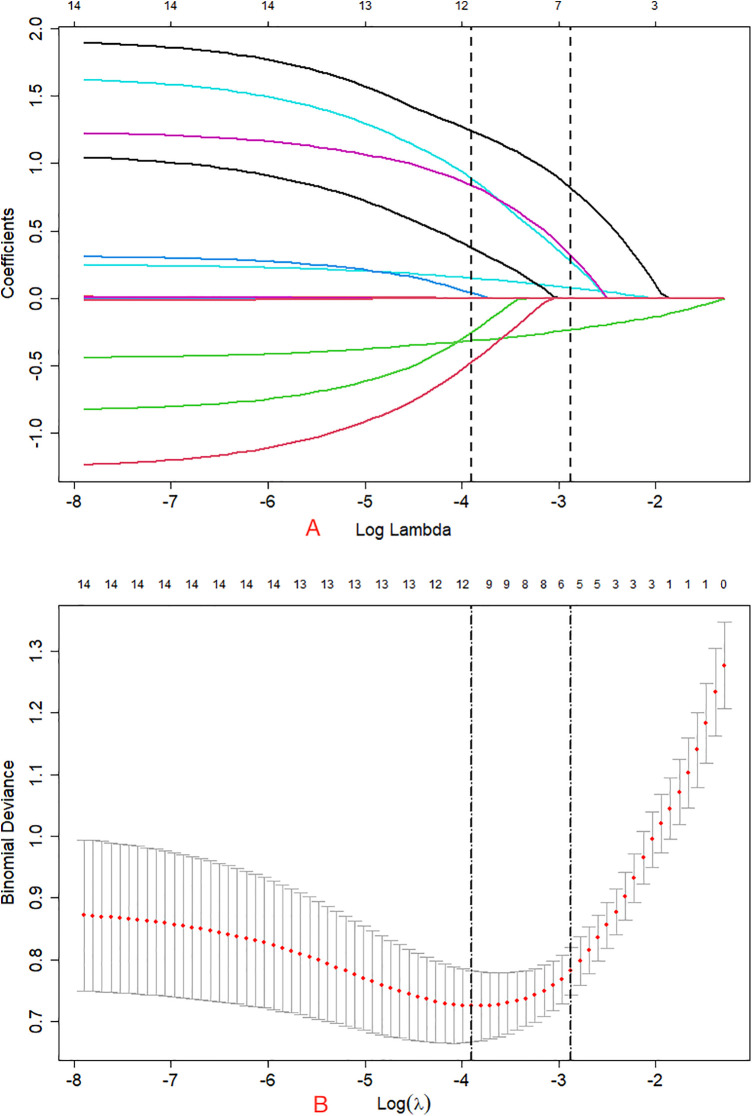
Screening variables by LASSO regression: **(A)** LASSO regression model graph. In this study, five non-zero coefficients were screened using Lambda.min as the criterion.; **(B)** Lambda (adjustment parameter) was obtained by cross-validated LASSO regression. The left dashed line is Lambda at minimum error (Lambda.min), the right dashed line is Lambda at standard error (Lambda.1-SE).

On the basis of clinical experience and previous studies, a total of 30 indicators were collected for each patient. Based on univariate logistic regression analysis, we identified 13 risk factors with statistical significance in the training cohort (Table 2). To reduce noise, variables that were not significant in the univariate logistic regression analysis were not included in lasso regression. Subsequently, we included 13 potential predictor variables in the LASSO regression analysis, which helped prevent overfitting of the variables and improve the accuracy of the prediction model. According to 1-SE of the minimum standard of non-zero coefficients, finally 5 variables were retained (Figure 1). The selected features included ALB, Jumping lesions (14, 15), Psoas abscess, Bone graft materials CRP normalization days (Table 3).

**Table 2:** Univariate analysis of patients and variables for STB cases.

Variables	Union group	Nonunion group	OR	CI	*P*
Age	51 (42, 62)	57.5 (45.5, 66.75)	1.029	1.029 (1.008–1.053)	<0.05
ALB	36.65 (34.48, 40.42)	29.7 (28.63, 32.38)	0.66	0.66 (0.576–0.742)	<0.001
HB	118.2 ± 17.36	103.91 ± 15.57	0.95	0.95 (0.928–0.97)	<0.001
Pre op CRP	10.25 (4.95, 22.48)	21.6 (13.45, 37.28)	1.023	1.023 (1.007–1.04)	<0.01
Pre op ESR	57 (38, 68)	67 (55, 72)	1.018	1.018 (1.002–1.036)	<0.05
CRP normalization days	7 (6, 10)	11 (8, 14)	1.163	1.163 (1.083–1.258)	<0.001
Post op Drainage	485.49 ± 222.99	585.09 ± 300.01	1.002	1.002 (1–1.003)	<0.05
Bone Graft Materials			5.377	5.377 (2.759–10.75)	<0.001
Autografts	92 (77)	22 (38)			
Synthetic materials	28 (23)	36 (62)			
Bone Grafting Techniques		0.396	0.396 (0.168–0.858)	<0.05	
Posterior	82 (68)	49 (84)			
Anterior	38 (32)	9 (16)			
OP > 3 h			3.143	3.143 (1.592–6.502)	<0.01
No	60 (50)	14 (24)			
Yes	60 (50)	44 (76)			
Psoas Abscess			3.463	3.463 (1.677–7.264)	<0.001
No	102 (85)	36 (62)			
Yes	18 (15)	22 (38)			
Jumping lesions			2.925	2.925 (1.362–6.355)	<0.01
No	104 (87)	40 (69)			
Yes	16 (13)	18 (31)			
AID			6.24	6.24 (1.726–29.38)	<0.01
No	117 (98)	50 (86)			
Yes	3 (2)	8 (14)			

**Table 3:** Variables according to 1-SE of the minimum standard of non-zero coefficients’.

Variables	OR	CI	Z	*P*
ALB	0.636	0.636 (0.541–0.748)	−5.494	<0.001
CRP normalization days	1.276	1.276 (1.134–1.436)	4.055	<0.001
Bone Graft Materials	7.62	7.620 (2.575–22.554)	3.668	<0.001
Psoas Abscess	3.665	3.665 (1.178–11.404)	2.243	<0.05
Jumping lesions	4.625	4.625 (1.364–15.679)	2.458	<0.05

### Nomogram construction

**Figure 2:**
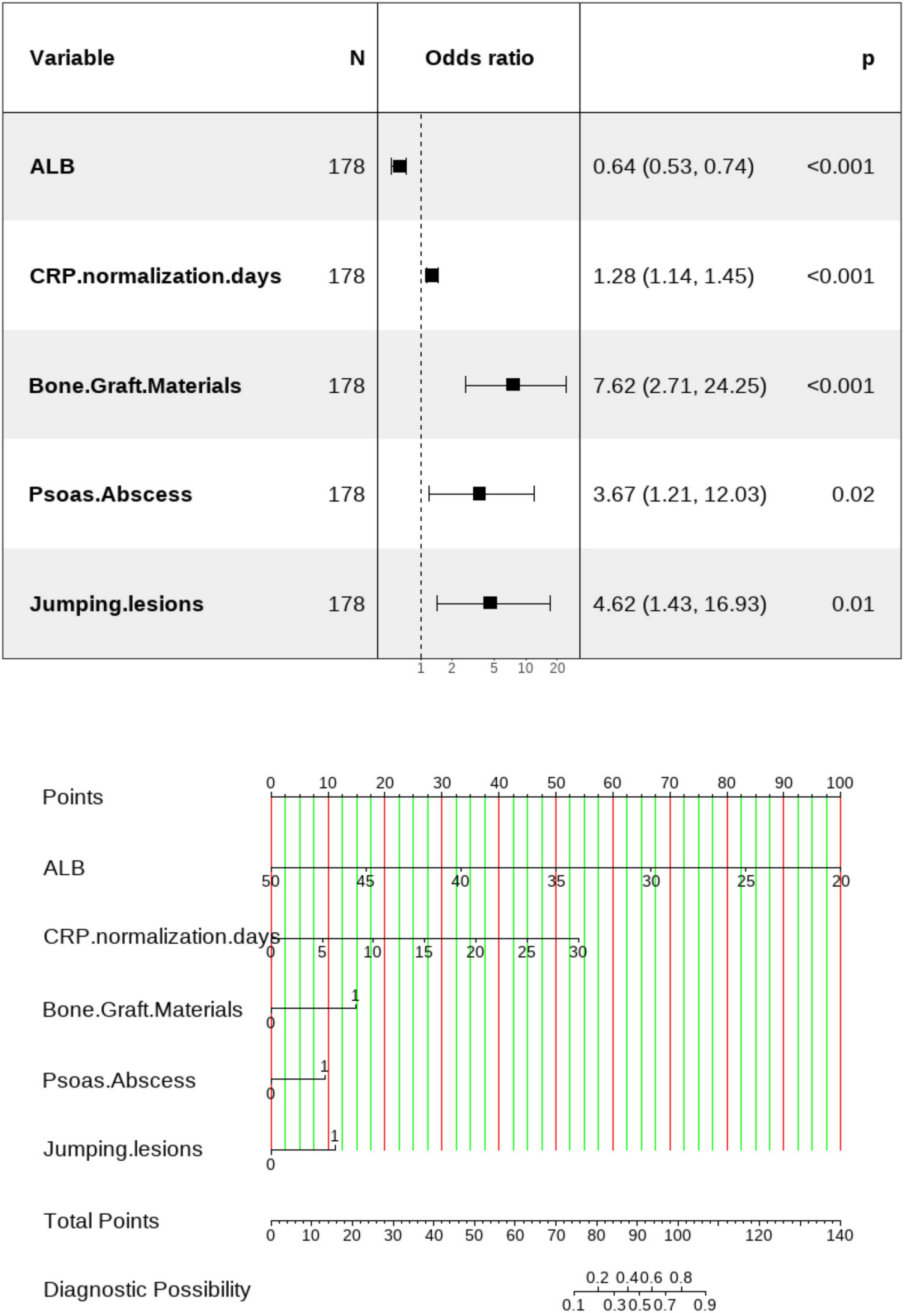
Forest plot of multivariable logistic regression for variables and nomogram scoring method for postoperative bone nonunion in STB patients. The corresponding score (top line) is found according to the value of each predictor variable (the line after each variable), then the values of the individual scores are summed to obtain the total score, and the corresponding predicted probability is based on the total score (bottom line).

Based on the one-standard-error (1-SE) criterion for selecting nonzero coefficients in LASSO regression, a nomogram was developed to differentiate between Union and Nonunion (Figure 2). In the nomogram, there were corresponding scores for each predictor variable according to the rating scale. The specific scores of all the variables were subsequently summed to obtain the total points. Overall, the probability of the two groups was calculated by means of drawing a vertical line downward at the total points.

### Performance and validation of the nomogram

The receiver operating characteristic curve showed that the AUC of the nomogram were 0.947 (95% confidence interval, 0.915–0.978).

**Figure 3:**
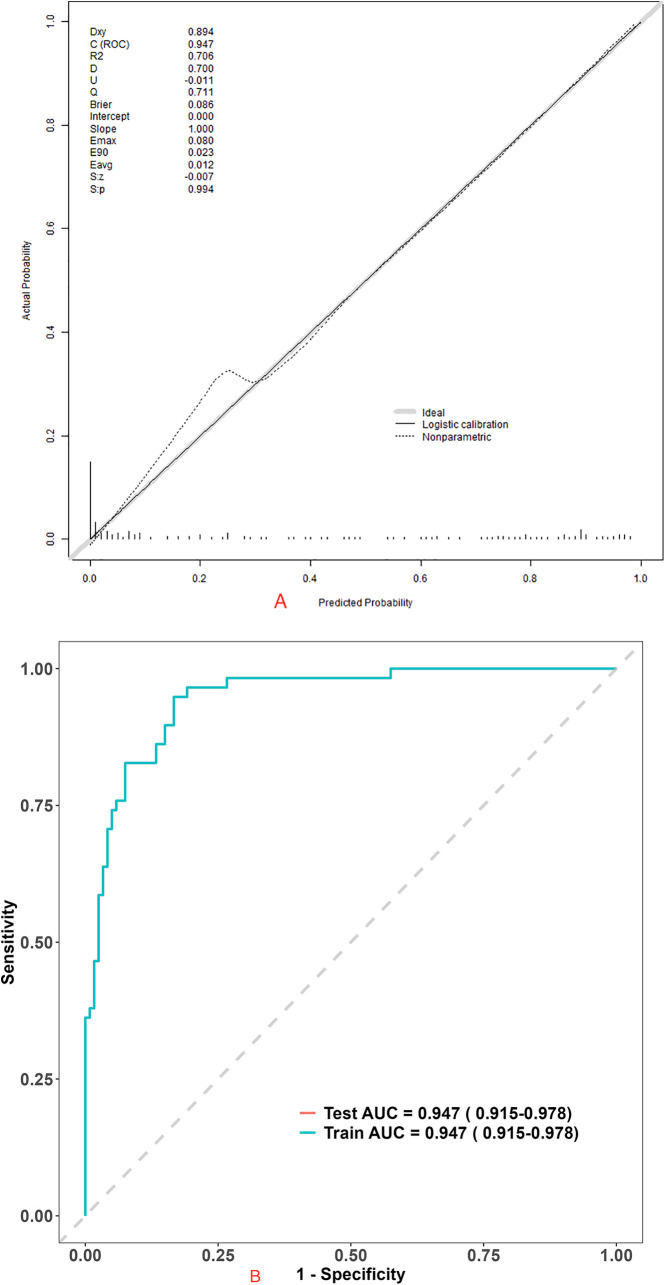
Evaluation of nomogram clinical prediction model: **(A)** calibration curve of the model; **(B)** ROC curve of the model, AUC were 0.947 (95% confidence interval, 0.915–0.978).

In addition, The Hosmer-Lemeshow test indicated good model calibration (*p* = 0.392). In the internal validation using 500 bootstrap resamples, the calibration curve showed high consistency between the predicted and actual findings (Figure 3).

**Figure 4:**
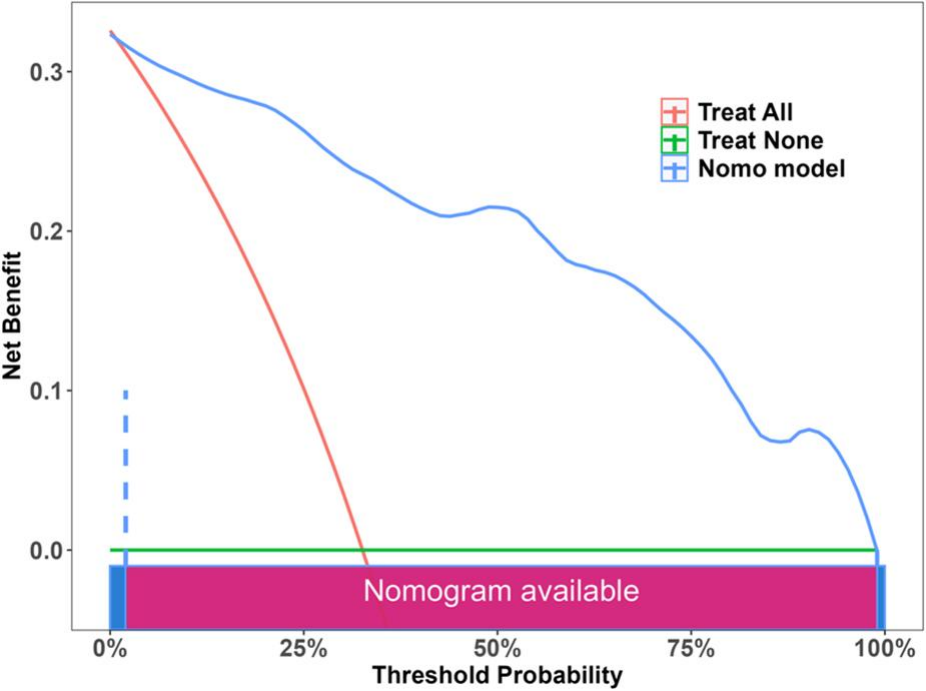
Decision curve analysis of the nomogram, the greatest clinical net benefit in predicting bone nonunion if the line graph was within the threshold range of 0.02 to 0.99.

The DCA results indicated that the nomogram provided the greatest clinical net benefit in predicting bone nonunion if the line graph was within the threshold range of 0.02 to 0.99 (Figure 4). This result indicates the clinical practicability of the nomogram.

## Discussion

This study successfully developed and internally validated the first multivariate nomogram specifically designed to predict postoperative bone nonunion risk in patients undergoing instrumented fusion for spinal tuberculosis. The model demonstrated exceptional discriminative ability 0.947 (95% confidence interval, 0.915–0.978) and robust calibration, indicating high accuracy and reliability. Its performance significantly surpasses existing predictive tools not specifically validated in infected cohorts and incorporates critical infection-related variables previously overlooked. The nomogram's strength lies in its integration of five readily available preoperative predictors, ALB, Jumping lesions, Psoas abscess, Bone graft materials, and CRP normalization days, providing clinicians with a practical, individualized risk assessment tool prior to surgery.

Consistent with previous studies in non-infected surgeries, we confirmed the prognostic significance of nutritional status and inflammatory markers. For instance, Wilson et al. (15) reported that hypoalbuminemia (ALB < 35 g/L) was associated with a 3.5-fold increase in nonunion risk (OR = 3.5, 95% CI: 1.6–7.8), a finding corroborated in our infected cohort. Similarly, Chen (16) highlighted the predictive value of CRP dynamics, showing that prolonged CRP elevation significantly correlated with nonunion in non-infectious bone surgery. These consistencies reinforce the biological plausibility of albumin and CRP as universally important factors in bone healing, irrespective of infection status.

Second, differences in sample composition and database sources may account for variations in predictor selection. While established models frequently emphasize metabolic factors such as diabetes, smoking, and osteoporosis, these did not retain significance in our final model (6, 7). This suggests that in the setting of active spinal tuberculosis, acute infectious and inflammatory processes may overshadow the influence of chronic metabolic comorbidities. Moreover, ethnic and regional differences, such as the high prevalence of spinal tuberculosis in endemic areas, may further limit the generalizability of non-infection-based models.

The type of bone graft material emerged as another crucial predictor, likely due to differences in osteoconductivity, osteoinductivity, and immune compatibility in an infected milieu. Autografts are considered the gold standard (17), synthetic materials may exhibit higher failure rates in tuberculosis settings, nuance that common non-infection models do not address.

Another notable distinction lies in the model's sensitivity and specificity. Our nomogram achieved high discriminative performance, which exceeds values typically reported in non-infected spinal fusion models. This improvement likely stems from the inclusion of highly specific infection-related predictors. For example, the use of CRP normalization days provides a dynamic assessment of treatment response that is particularly relevant in infected cases. The presence of psoas abscesses and Jumping lesions also adds severity information that enhances predictive accuracy.

Although the nomogram demonstrated excellent discriminative performance, several issues warrant cautious interpretation. Firstly, this study did not include data on drug resistance patterns, which are known to affect the clinical course and treatment outcomes in STB. Drug resistance, particularly multidrug-resistant or extensively drug-resistant strains, is associated with delayed lesion resolution, more prolonged therapy, and poorer outcomes, and inadequate eradication may impair the local environment necessary for bone healing (18). Therefore, the absence of drug-resistance data requires cautious interpretation; multidrug-resistant or extensively drug-resistant strains are known to delay lesion resolution and impair bone healing. Future nomograms should incorporate drug susceptibility testing to enhance predictive accuracy.

Additionally, nutritional status was assessed only through serum albumin, without considering broader indicators (e.g., prealbumin, vitamin D, or body mass index) that affect tissue repair. Malnutrition and low protein status are well-recognized risk factors for impaired wound and bone healing, as essential nutrients are critical for osteogenesis, collagen synthesis, and immune function (19). Finally, Socioeconomic factors, such as income, education, and access to follow-up care, were also not captured, despite their known impact on musculoskeletal healing. These factors may affect adherence to prolonged anti-tuberculosis therapy and postoperative rehabilitation, potentially leading to delayed union and reduced success rates (20). These unmeasured variables may represent important confounders in postoperative bone fusion outcomes.

Despite the promising performance of the proposed nomogram, several issues warrant cautious interpretation. This study was conducted at a single tertiary referral center and employed a retrospective design, which may introduce selection bias and limit generalizability to broader patient populations. In addition, although internal validation was performed, the sample size remains relatively limited. Therefore, external validation in larger, prospective, multicenter cohorts is essential to confirm the robustness, reproducibility, and clinical applicability of this prediction model before routine implementation.

## Advantages and limitations

This study has several strengths. It is the first to develop a nomogram specifically for predicting postoperative bone nonunion in spinal tuberculosis, incorporating infection-related predictors such as CRP normalization time. In addition, the use of a comprehensive institutional database with standardized surgical and medical management enhances internal validity.

Nevertheless, several limitations must be acknowledged. The retrospective, single-center design inherently limits external validity and may introduce selection bias. The relatively small sample size increases the risk of model optimism despite internal validation. Moreover, the absence of data on drug resistance, comprehensive nutritional status, and socioeconomic factors may restrict the model's completeness. Therefore, multicenter, prospective studies with external validation are essential before widespread clinical application.

## Conclusion

In conclusion, this study developed an exploratory, internally validated nomogram for estimating the risk of postoperative bone nonunion in patients undergoing instrumented fusion for spinal tuberculosis. While the model demonstrates promising predictive performance, its clinical application should be considered preliminary. Although internal validation was performed, external validation in larger, prospective, multicenter cohorts is needed to confirm the robustness and clinical applicability of the prediction model.

## Data Availability

The original contributions presented in the study are included in the article/Supplementary Material, further inquiries can be directed to the corresponding author/s.
